# Surface Molecules Released by *Trypanosoma cruzi* Metacyclic Forms Downregulate Host Cell Invasion

**DOI:** 10.1371/journal.pntd.0004883

**Published:** 2016-08-02

**Authors:** Tatiana Mordente Clemente, Cristian Cortez, Antônio da Silva Novaes, Nobuko Yoshida

**Affiliations:** 1 Departamento de Microbiologia, Imunologia e Parasitologia, Universidade Federal de São Paulo, São Paulo, Brasil; 2 Departamento de Medicina, Universidade Federal de São Paulo, São Paulo, Brasil; McGill University, CANADA

## Abstract

**Background:**

The question whether metacylic trypomastigote (MT) forms of different *T*. *cruzi* strains differentially release surface molecules, and how they affect host cell invasion, remains to be fully clarified. We addressed that question using *T*. *cruzi* strains that differ widely in the ability to invade cells.

**Methodology/Principal Findings:**

Metacyclic forms were incubated at 37°C for 1 h in complete D10 medium or in nutrient-deprived PBS containing Ca^2+^ and Mg^2+^ (PBS^++^). The conditioned medium (CM), collected after parasite centrifugation, was used for cell invasion assays and Western blot analysis, using monoclonal antibodies directed to gp82 and gp90, the MT surface molecules that promote and negatively regulate invasion, respectively. CM of poorly invasive G strain (G-CM) contained high amounts of gp90 and gp82, either in vesicles or as soluble molecules. CM of highly invasive CL strain (CL-CM) contained gp90 and gp82 at very low levels. HeLa cells were incubated for 1 h with CL strain MT in D10, in absence or in the presence of G-CM or CL-CM. Parasite invasion was significantly inhibited by G-CM, but not by CL-CM. As G strain MT invasion rate in D10 is very low, assays with this strain were performed in PBS^++^, which induces invasion-promoting lysosome-spreading. G-CM, but not CL-CM, significantly inhibited G strain internalization, effect that was counteracted by preincubating G-CM with an anti-gp90 monoclonal antibody or anti-gp82 polyclonal antibody that do not recognize live MT. G strain CM generated in PBS^++^ contained much lower amounts of gp90 and gp82 as compared to CM produced in D10, and exhibited lower inhibitory effect on host cell invasion.

**Conclusion/Significance:**

Our data suggest that the surface molecules spontaneously released by MT impair parasite-host cell interaction, gp82 presumably competing with the molecule expressed on MT surface for the host cell receptor, and gp90 further contributing to down modulate invasion.

## Author Summary

The molecular basis of wide differences in the ability of metacylic trypomastigote (MT) forms of different *T*. *cruzi* strains to invade host cells is not fully understood. We investigated the possibility that surface molecules gp82 and gp90, which function as mediator and down regulator of cell invasion respectively, were differentially released by different strains and affected MT internalization. Conditioned medium of poorly invasive G strain (G-CM) and highly invasive CL strain (CL-CM) was tested in MT invasion assays. Western blot analysis revealed high levels of gp82 and gp90 in G-CM, either in vesicles or as soluble molecules, and low levels in CL-CM. Invasion of human HeLa cells by MT of both strains was inhibited in the presence of G-CM, but not of CL-CM. Incubation of G-CM with anti-gp90 or anti-gp82 antibodies that do not recognize live MT counteracted the inhibitory effect of G-CM. We infer from these data that gp90 and gp82 shed into medium are as relevant as the molecules expressed on MT surface in interacting with host cells and to regulate the invasion process.

## Introduction

Spontaneous release of *T*. c*ruzi* surface antigens as membrane vesicles was described more than two decades ago in a study using tissue culture trypomastigotes (TCT) [1], which are the *in vitro* counterparts of parasites circulating in the bloodstream. TCT vesicles would be carriers of virulence factors [2]. Injection into mice of TCT vesicles, enriched in surface glycoproteins of the gp85/trans-sialidase (TS) superfamily, prior to *T*. *cruzi* infection led to increased heart parasitism, an intense inflammatory response, severe heart pathology and an earlier death [3]. More recently, it was reported that vesicles from different parasite strains trigger differential innate and chronic immune responses [4]. As regards the metacyclic trypomastigote (MT) forms, which initiate the infection of the mammalian host, the major influence of MT-released molecules would be at the early stage of host cell invasion process, provided that MT residence in the mammalian host is transient, spanning the step of internalization through the escape to the cytoplasm.

Analysis of extracellular vesicles and soluble proteins shed by epimastigotes and MT of *T*. *cruzi* Dm28 clone has revealed populations enriched in larger vesicles, expected to be mainly plasma membrane-derived, and those enriched in smaller vesicles, supposed to be mainly derived from the exocytic fusion of multivesicular bodies with the flagellar pocket membrane [5]. MT-specific gp82, the surface molecule that mediates target cell adhesion/invasion [6], was shown to be shed as vesicles or soluble proteins [5]. The release of gp90, the MT-specific surface molecule that negatively regulates cell invasion [7], was not determined.

The wide difference in the ability of MT from different *T*. *cruzi* strains to invade host cells [8], such as observed in strains G and CL belonging to divergent genetic groups [9], has been associated with differential expression of surface molecules involved in cell adhesion, gp90 playing a determinant role [10]. Gp90 binds to host cells but, differently from gp82, is unable to trigger Ca^2+^ signal required for lysosome mobilization and exocytosis [10,11], an event crucial for the parasitophorous vacuole formation [12–14]. Highly invasive CL strain MT express gp90 at lower levels than poorly invasive G strain MT [8]. What remains to be fully clarified is the contribution of gp90 shed by MT for the observed differences. Here we investigated whether MT adhesins gp90 and gp82 were differentially released by G and CL strains and how they influenced parasite internalization. High amounts of gp90 and gp82 were shed by G strain MT, whereas the release of these molecules by CL strain MT molecules was minimal. The evidences are that gp90 and gp82 released by G strain MT contribute to the low invasion capacity of this parasite.

## Methods

### Parasites, mammalian cell culture and invasion assays

*T*. *cruzi* strains G and CL, belonging to genetic groups TcI and TcVI, respectively [15], were used. Parasites were maintained cyclically in mice and in liver infusion tryptose medium containing 5% fetal bovine serum. Metacyclic forms from cultures at the stationary growth phase were purified by passage through DEAE-cellulose column, as described [16]. Maintenance of human epithelial HeLa cells and monkey fibroblast Vero cells (purchased from Instituto Adolfo Lutz, São Paulo) and *T*. *cruzi* invasion assays are described elsewhere [17,18]. The multiplicity of infection was 20:1 for G strain and 10:1 for CL strain. Invasion assay were performed in DMEM with 10% fetal bovine serum (D10) or in PBS^++^ (PBS containing per liter: 140 mg CaCl_2_, 400 mg KCl, 100 mg MgCl_2_.6H_2_O, 100 mg MgSO_4_.7H_2_O, 350 mg NaHCO_3_). After 1 h incubation with parasites, the duplicate coverslips coated with HeLa or Vero cells were washed in PBS, fixed in Bouin solution, stained with Giemsa, and sequentially dehydrated in acetone, a graded series of acetone:xylol and xylol. The number of intracellular parasites was counted in 250 stained cells. Epimastigotes were harvested from cultures at the exponential growth phase and washed in PBS before use. Tissue culture trypomastigotes were obtained as follows: Vero cells were infected with MT. Starting on day six, the parasites released into the medium were collected.

### Ethics statement

Procedures using mice conformed with Brazilian National Committee on Ethics Research (CONEP) ethics guidelines, and the study was approved by Ethical Committee for animal experimentation of the Universidade Federal de São Paulo (Protocol No 0005/13).

### Preparation of parasite conditioned medium (CM) and fractionation

CM was prepared by incubating 10^8^ metacyclic forms at 37°C for 1 h in 0.1 ml D10 or PBS^++^. After centrifugation, the supernatant (conditioned medium) was collected, 0.1 ml of 0.5% NP-40 detergent solution containing protease inhibitor cocktail (cOmplete, Roche) was added to the pellet, the extract was centrifuged at 12,000 g for 5 min. The pellet was resuspended in PBS and used for Western blot analysis. After loading 10 μl of conditioned medium and detergent-soluble extract into 10% SDS-PAGE gel, followed by transfer to the nitrocellulose membrane, the gp90 and gp82 molecules were revealed using specific monoclonal antibodies. For cell invasion assays, the conditioned medium was diluted 1:50 in D10 or PBS^++^. Fractionation of the parasite conditioned medium followed the procedure described elsewhere [5]. Briefly, 10^8^ metacyclic forms were incubated at 37°C for 1 h in D10 medium, which contained serum centrifuged overnight at 100,000g to discard particles that could interfere with vesicle counting, or in PBS^++^. After centrifugation at 3,000g for 10 min at 4°C, the cell-free supernatant was filtered in 0.45-μm syringe filter (Millipore), transferred to Ultra-Clear tubes (5 ml, 13x51 mm), and centrifuged at 100,000g for 2 h at 4°C to obtain the first pellet, enriched in larger extracellular vesicles (V2). The resulting supernatant was transferred to another Ultra-Clear tube and then centrifuged at 100,000g for 16 h at 4°C, to obtain the second pellet enriched in smaller vesicles (V16) and vesicle-free supernatant (VF). All ultracentrifugation steps were carried out using a SW55Ti swinging-bucket rotor (Beckman Coulter) in an Optima L-100 XP ultracentrifuge (Beckman Coulter).

### Nanoparticle tracking analysis

The size of extracellular vesicles from metacyclic forms (V2 and V16) of *T*. *cruzi* strains G and CL was determined by nanoparticle tracking analysis (NTA), by measuring the rate of Brownian motion to particle size, using a Malvern NanoSight (NS300) system that tracks particles individually and using the Stokes-Einstein equation to calculate their diameters. Three replicates of diluted vesicles fraction aliquots (1 ml in PBS) were injected into the machine’s specimen chamber and vesicles were tracked and measured for 30 sec, three times for each sample, in a constant flow rate at RT.

### Scanning electron microscopy

Parasites were washed with PBS and attached to cover slips that were pretreated with 0.05% poly-L-lysine for 30 minutes, and washed in water. After 5 min centrifugation at 500 g for greater adherence of parasites, they were fixed for 1 h, at room temperature, with 2.5% glutaraldehyde in 0.1 M sodium cacodylate buffer, pH 7.2. The fixed parasites were washed four times with 0.1 M sodium cacodylate buffer and then post-fixed with 1% osmium tetroxide in the same buffer, at room temperature. Following washings with cacodylate buffer, 30 min treatment with 1% tannic acid in water, three washes in water, 30 min impregnation with 1% osmium tetroxide, three washes in water, the samples were subjected to a gradual dehydration in a series of ethanol solutions, and drying at the critical point apparatus using CO_2_. After assembly in support of the SEM sample holder (stub) using Superglue, the material was coated with gold by sputtering, and observed in Scanning Electron Microscope.

### Flow cytometry and indirect immunofluorescence assays

Live metacyclic trypomastigotes (1x10^7^) were incubated on ice for 1 h with monoclonal antibody 3F6 or 1G7, directed respectively to MT-specific surface molecule gp82 or gp90. Thereafter, the parasites were fixed with 4% para-formaldehyde for 20 min. Following washings in PBS, the parasites were incubated with Alexa Fluor 488-conjugated anti-IgG for 1 h at room temperature and the number of fluorescent parasites was estimated with a Flow Cytometer. Control parasites were incubated with the secondary antibody only. Images of fluorescent parasites were acquired on a fluorescence microscope coupled to a digital camera. To visualize HeLa cell lysosomes, coverslips with adherent cells were incubated for 30 min at 37°C in D10 or in PBS^++^, or were incubated with parasites in D10 for 1 h. After fixation with 4% para-formaldehyde in PBS for 30 min, the cells were treated with 50 mM NH_4_Cl in PBS for 30 min and washed in PBS. The cells were then incubated for 1 h at room temperature with mouse anti-human LAMP2 diluted 1:8 (v/v) in a PBS solution containing 0.15% gelatin, 0.1% sodium azide and 1% saponin (PGN-Saponin). After washings in PBS, the coverslips were incubated for 1 h with Alexa Fluor 488-conjugated anti-mouse IgG (Invitrogen), diluted 1:300 in PGN-Saponin containing 500 ng/ml phalloidin-TRITC and 10 μg/ml DAPI (4′,6′-1-diamino-2-phenylindole dihydrochloride), followed by washes in PBS and subsequent mounting of coverslips in ProLong Gold (Invitrogen). Confocal images were acquired in a Leica TCS SP8 laser-scanning microscope (Leica, Germany) using an oil immersion Plan-Apochromat 63X objective (numerical aperture 1.4). The series of images obtained from confocal z-stacks were processed and analyzed using Leica LAS AF (Leica, 2012, Germany) and Imaris (Bitplane) software.

### Preparation of native and recombinant MT proteins and cell binding assays

Gp90 was purified from detergent soluble extract of G strain MT, by affinity chromatography on immobilized anti-gp90 monoclonal antibody 1G7, as described [19]. The recombinant gp90 protein, containing the C-terminal domain of *T*. *cruzi* gp90 (GenBank accession number L11287), and the recombinant protein containing the full-length gp82 sequence (GenBank accession number L14824) were produced in *Escherichia coli* as described [20,21]. All steps for the purification of the recombinant proteins followed a previously described procedure [21], and the purified protein was analyzed by Coomassie blue staining of SDS-PAGE gel and by immunoblotting using monoclonal antibody directed to gp90 or gp82. For cell binding assay, HeLa cells were fixed with 4% para-formaldehyde in PBS for 30 min, washed and blocked with PBS containing 2 mg/ml BSA (PBS/BSA). Following 1 h incubation with the native or recombinant gp90, the cells were incubated sequentially with anti-gp90 monoclonal antibody 1G7, and peroxidase-conjugated anti-mouse IgG, all diluted in PBS/BSA. The final reaction was revealed by *o*-phenilenediamine and the absorbance at 492 nm read in a Multiscan MS ELISA reader.

### Statistical analysis

The Student’s *t* test, as implemented in GraphPad Prism software (Version 6.01), was employed.

## Results

### MT conditioned medium containing high gp90 and gp82 levels inhibits invasion of human epithelial cells by *T*. *cruzi*

**Fig 1:**
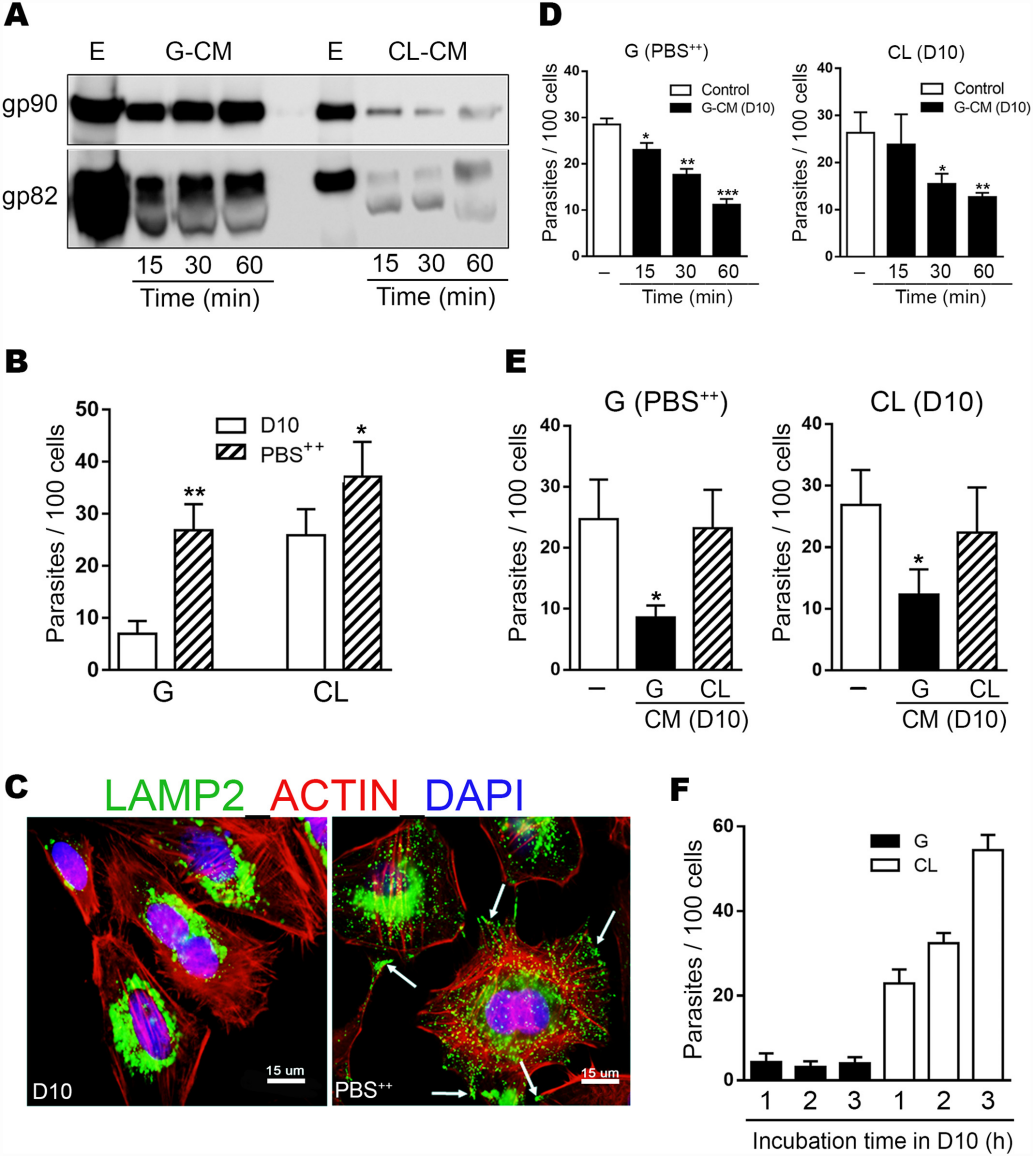
Effect of *T*. *cruzi* conditioned medium containing high gp90 and gp82 amounts on host cell invasion by metacyclic forms. A) Western blot of detergent-soluble MT extract (E) of G and CL strains and the corresponding conditioned medium (CM) generated at 15, 30 and 60 min incubation of MT in D10, was probed with anti-gp90 mAb 5E7 or anti-gp82 mAb 3F6. B) HeLa cells were incubated for 1 h with G or CL strain MT, in PBS^++^ or in D10. After fixation and staining with Giemsa, the number of intracellular parasites was counted in a total of 250 cells. Values are the means ± SD of five independent assays performed in duplicate. MT invasion rate was significantly higher in PBS^++^ than in D10 (*P< 0.*05*, **P<0.0001). C) HeLa cells were incubated for 30 min in D10 or in PBS^++^, and then processed for immunofluorescence analysis using anti-LAMP2 antibody, Alexa Fluor 488-conjugated anti-mouse IgG (green), phalloidin-TRITC (red) for actin visualization and DAPI (blue) for DNA. Epifluorescence microscope, with 100X objective, was used. Scale bar: 15 μm. Note the lysosome scattering and LAMP2 accumulation (arrow) at the edges of cells incubated in PBS^++^. D) HeLa cells were incubated for 1 h with G or CL strain MT, in PBS^++^ or in D10, in absence or in the presence of G-CM generated as in (A), at 1:50 dilution. After fixation and staining with Giemsa, the number of intracellular parasites was counted in a total of 250 cells. Values are the means ± SD of three independent assays performed in duplicate. G-CM significantly inhibited cell invasion by G strain MT (*P<0.01, ** *P* = 0.0005, *** *P*<0.0001) and by CL strain MT (*P<0.05, ** *P*<0.01). E) HeLa cells were incubated for 1 h with G or CL strain MT, in PBS^++^ or in D10, in absence of in the presence of G-CM or CL-CM generated after 1 h MT incubation in D10, and processed for parasite counting. Values are the means ± SD of four independent assays performed in duplicate. Invasion by both strains was significantly inhibited by G-CM (*P<0.005) but not by CL-CM. F) HeLa cells were incubated for the indicated times with G or CL strain MT in D10 and processed for intracellular parasite counting. Invasion by CL strain, but not by G strain, increased proportional to the incubation times.

**Fig 2:**
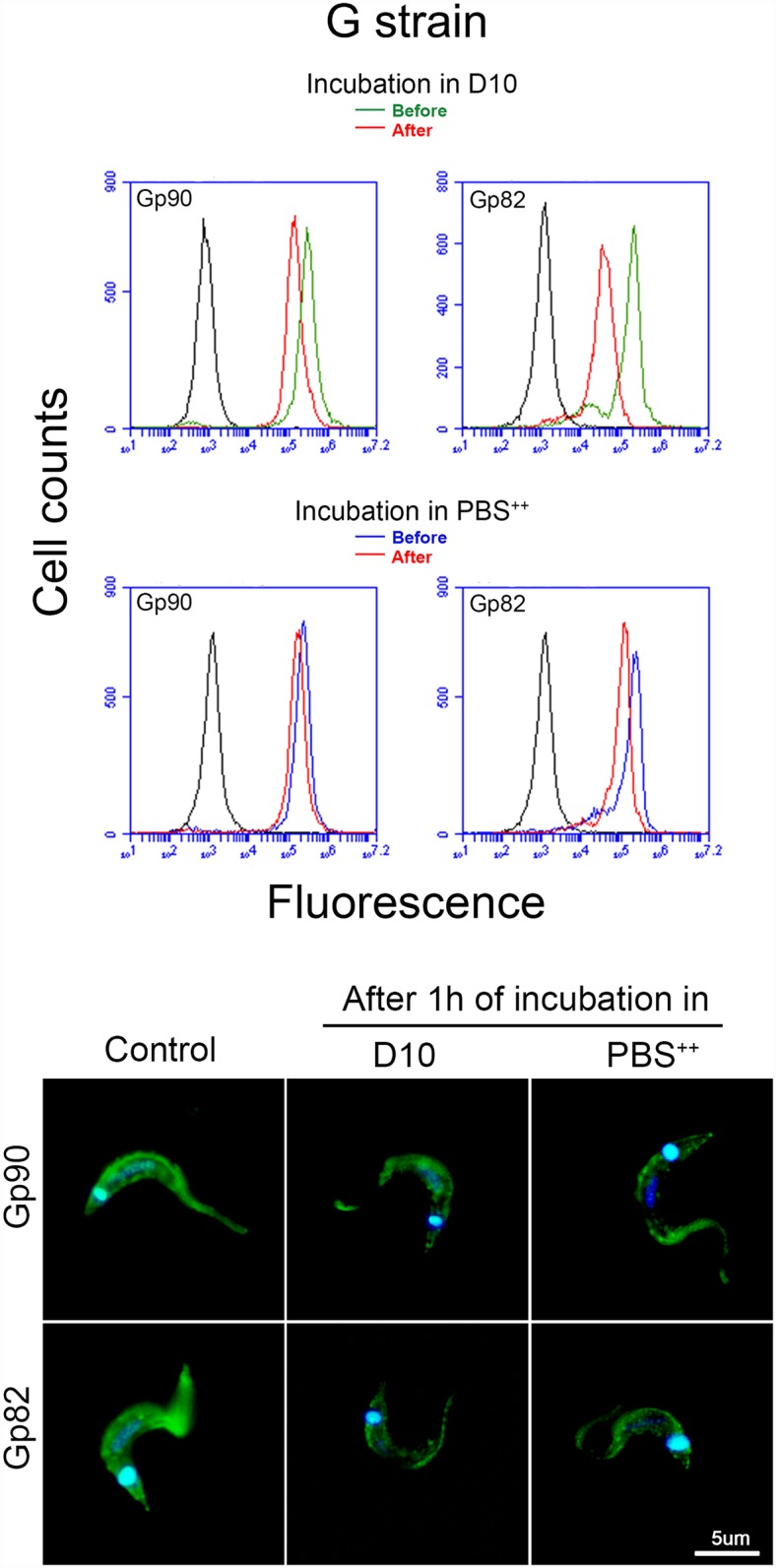
Change in the expression of surface gp90 and gp82 proteins in *T*. *cruzi* G strain metacyclic forms after incubation in different media. Parasites were incubated or not in D10 or PBS^++^ for 1h at 37°C. After centrifugation, the supernatant was discarded and the parasites were incubated for 1 h with monoclonal antibody directed to gp90 or gp82. Following fixation and reaction with Alexa Fluor 488-conjugated anti-IgG, the parasites were analyzed by flow cytometry. Controls consisted of parasites incubated with the second antibody only. Shown in the lower panel are images of parasites visualized by epifluorescence microscope, with 100X objective.

We examined the release of MT surface molecules gp90 and gp82 by G and CL strains and its effect on host cell invasion. Metacyclic forms were incubated in full nutrient D10 medium for 15, 30 or 60 min, and the conditioned medium (CM) obtained after removal of parasites was analyzed by Western blot for detection of gp90 and gp82 molecules. High amounts of gp90 and gp82 were released into medium by G strain MT as early as 15 min after incubation (Fig 1A). By contrast, gp90 and gp82 were shed at low levels by CL strain MT, even after 60 min incubation in D10 (Fig 1A). Next, the effect of conditioned medium from G strain (G-CM) on MT invasion of human epithelial HeLa cells was determined. Cell invasion assays with G strain were performed in nutrient-deprived PBS^++^ because its invasion rate is very low in D10 (Fig 1B). In PBS^++^, G strain MT entered HeLa cells at a rate similar to that of CL strain MT in D10 (Fig 1B). The increased susceptibility of HeLa cells to MT invasion in PBS^++^ has been associated with PBS^++^-triggered lysosome spreading that culminates in exocytosis [11], an event that contributes to the parasitophorous vacuole formation [12–14]. In HeLa cells incubated in D10 and processed for immunofluorescence, we visualize lysosomes concentrated in the perinuclear area (Fig 1C). After a 30 min incubation in PBS^++^, lysosomes spread toward the cell periphery and exhibited a propensity to accumulate at the cell edges (Fig 1C), which are the preferential sites of *T*. *cruzi* invasion [22]. HeLa cells were incubated for 1 h with G strain MT in PBS^++^ or with CL strain MT in D10, in the presence of G-CM at 1:50 dilution. In all experiments the same amount of D10 media was added to the controls, to rule out the possibility of any eventual effect of the small amount of D10. The invasion rate of both strains was significantly inhibited by G-CM, the highest effect being observed with G-CM obtained after 60 min incubation (Fig 1D). We also checked the effect of strain CL-CM on MT internalization. CL-CM had no significant inhibitory effect on G or CL strain entry into HeLa cells (Fig 1E). As the high shedding activity of G strain was detected within 15 min incubation in D10 (Fig 1A), we reasoned that incubation with host cells for times longer than 1 h would not increase parasite invasion. As predicted, the rate of G strain invasion after 1, 2 or 3 h incubation with HeLa cells in D10 was comparable, whereas invasion by CL strain MT with low shedding activity increased correspondingly with longer incubation times (Fig 1F). To determine the levels of gp90 and gp82 on MT surface after release of these molecules to extracellular medium, we analyzed G strain MT by flow cytometry before and after 1 h incubation in D10 or in PBS^++^. FACS analysis showed a diminished expression of gp90 and gp82 after MT incubation in D10, and a less pronounced reduction after incubation in PBS^++^ (Fig 2). The decrease in the expression of gp90 and gp82 after MT incubation in D10 or PBS^++^ was also detected in parasites processed for immunofluorescence (Fig 2). In the case of CL strain, the difference in gp82 and gp90 expression before and after incubation in D10 and in PBS^++^ was minimal (S1 Fig).

Conditioned medium from other developmental forms of G strain was also tested for the ability to inhibit MT invasion. HeLa cell invasion by both G and CL strain MT was inhibited by CM from tissue culture trypomastigotes (TCT), albeit to a lesser degree than G-CM, whereas epimastigote CM was without effect (S2A Fig). We examined whether molecules recognized by antibodies directed to gp90 or gp82 were present in CM from TCT, using CM from MT as control. The Western blot analysis revealed in CM from TCT a weak 90 kDa band recognized by anti-gp90 polyclonal antibody but not by mAb 1G7 or mAb 5E7, and doublet bands of around 82 kDa recognized by anti-gp82 polyclonal antibody but not by mAb 3F6 (S2B Fig).

**Fig 3:**
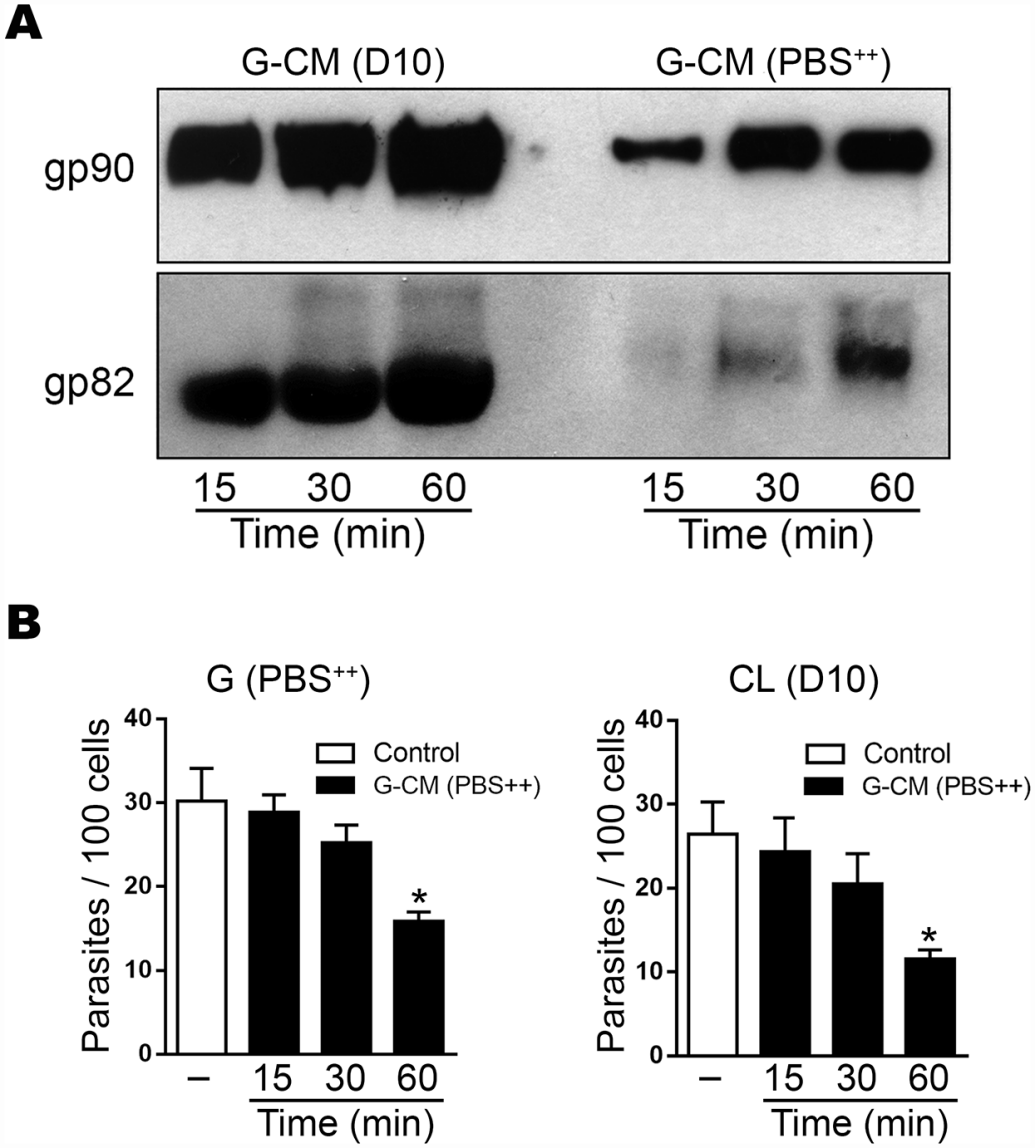
Differential release of gp90 and gp82 molecules by G strain MT in D10 and PBS^++^. A) Western blot of conditioned medium generated at 15, 30 and 60 min incubation of MT in D10 or PBS^++^ was probed with anti-gp90 or anti-gp82 mAb. Note the smaller amounts of gp90 and gp82 released in PBS^++^ as compared to D10. B) HeLa cells were incubated for 1 h with G or CL strain MT, in PBS^++^ or in D10, in absence of in the presence of G-CM generated in PBS^++^. After fixation and Giemsa staining, number of intracellular parasites was counted in a total of 250 cells. Values are the means ± SD of three independent assays performed in duplicate. G-CM generated at 60 min significantly inhibited cell invasion by both strains (*P<0.01).

Another question to be examined was whether G strain MT differentially released surface molecules into D10 and PBS^++^. Metacyclic forms were incubated in PBS^++^ for 15, 30 or 60 min, and the conditioned medium obtained after removal of parasites was analyzed by Western blot for detection of gp90 and gp82 molecules. Much lower amounts of gp90 and gp82 were shed in PBS^++^ as compared to D10 (Fig 3A). The effect of conditioned medium generated in PBS^++^ on MT invasion was determined. HeLa cells were incubated for 1 h with G strain MT in PBS^++^ or with CL strain MT in D10, in absence or in the presence of G-CM at 1:50 dilution. The invasion rate of both strains was significantly inhibited by G-CM obtained after 60 min incubation, but not by G-CM from 30 min incubation, which contained lower amounts of gp82 (Fig 3B).

### Gp90 and gp82 are released by G strain MT in extracellular vesicles and as soluble proteins

**Fig 4:**
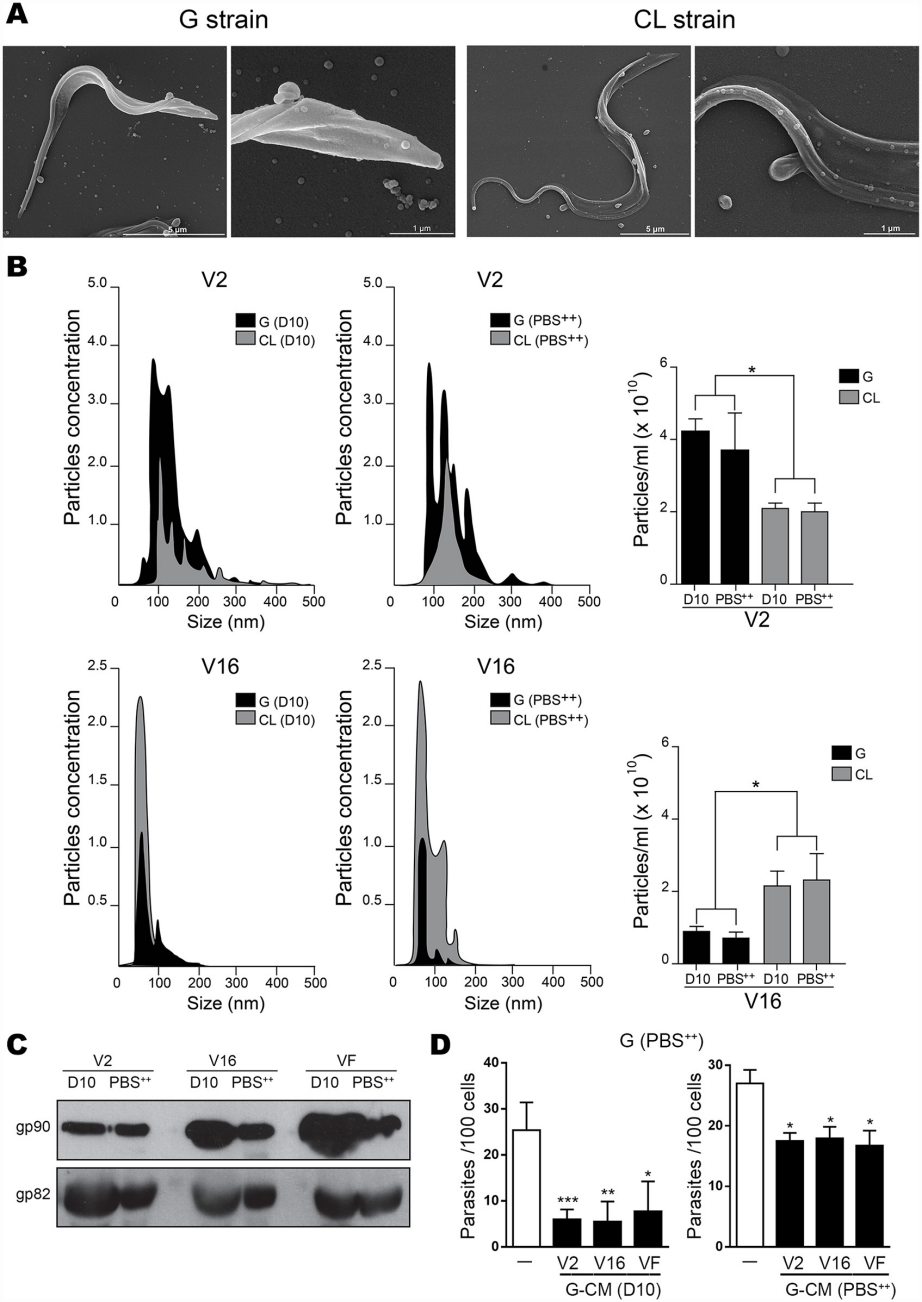
Release of vesicles by *T*. *cruzi* metacyclic tripomastigotes. A) Parasites were washed in PBS and processed for analysis by scanning electron microscopy. Scale-bars: 1–5 μm). B) After 1 h incubation in particle-free D10 medium or in PBS^++^, the parasites were centrifuged and the supernatant collected for fractionation. Large particles (V2) and smaller particles (V16), obtained after 2 h and 16 h ultracentrifugation respectively, were quantified by nanoparticle tracking analysis. Shown is the particle size distribution in conditioned medium from G and CL strain in D10 and PBS^++^. Data are representative of 3 independent experiments. The number of particles released by G and CL strains was significantly different (*P<0.05). C) The fractions V2, V16 and vesicle-free (VF) fractions obtained from G-CM were analyzed by Western blot using monoclonal antibodies directed to gp90 and gp82. D) HeLa cells were incubated for 1 h with G strain MT in PBS^++^ or in PBS^++^ plus V2, V16 or VF fractions of G-CM generated in D10 or PBS^++^. Data are representative of 3 independent experiments (*P<0.05, **P<0.01, ***P<0.005).

We examined the release of vesicles by MT of G and CL strains, by preparing parasites for scanning electron microscopy. Both strains released vesicles of various sizes (Fig 4A). To separate the extracellular vesicles according to their size, we followed the ultracentrifugation protocol described by Santos-Bayer et al. [5]. The distribution of vesicles, obtained after 2 h (V2) and 16 h (V16) ultracentrifugation of G-CM and CL-CM generated in D10 or in PBS^++^, is shown in Fig 4B. Regardless of the medium, vesicles of large size (V2) were found in higher numbers in G-CM than in CL-CM. By contrast, vesicles of smaller size (V16) were more abundant in CL-CM than in G-CM (Fig 4B). We examined the presence of gp90 and gp82 in V2, V6 and in vesicle-free (VF) fractions of G-CM by Western blot. Gp90 and gp82 were found in all fractions, and at higher levels in CM generated in D10 as compared to PBS^++^ (Fig 4C). The effect of the fractions from G-CM generated in D10 and PBS^++^ on MT invasion was examined. Fractions from G-CM generated in D10 exhibited higher inhibitory effect than G-CM generated in PBS^++^ (Fig 4D). The various fractions of CL-CM generated in D10 and in PBS^++^ were also analyzed. Gp82 was detected in fractions V2, V16 and VF from CM generated in D10 but not in CM generated in PBS^++^. Differently from G-CM, gp90 was not detected in CL-CM generated in D10 or PBS^++^. When tested in MT invasion assays, the various CL-CM fractions failed to show significant inhibitory effect.

### MT-shed gp90 and gp82 molecules down modulate host cell invasion

**Fig 5:**
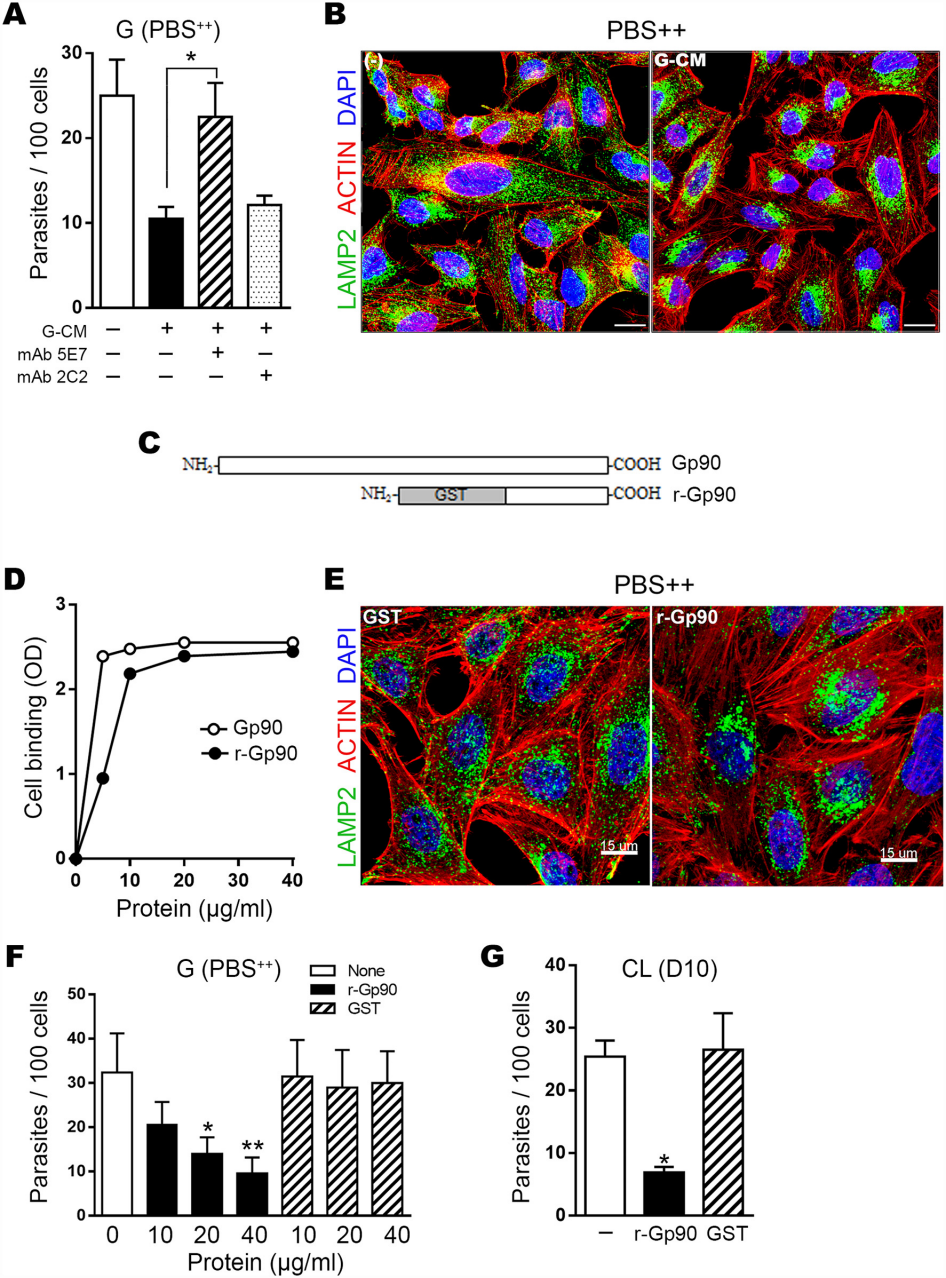
Inhibitory effect of gp90 molecule on PBS^++^–induced lysosome spreading and host cell invasion by MT. A) HeLa cells were incubated for 1 h in PBS^++^ with G strain MT in absence or in the presence of G-CM generated in D10 alone, or preincubated with anti-gp90 mAb 5E7 or unrelated mAb 2C2. After fixation and Giemsa-staining, the number of intracellular parasites was counted in 250 cells. Values are the means ± SD of three independent assays performed in duplicate. The inhibitory effect of G-CM was significantly reverted by mAb 5E7 (*P<0.005). B) HeLa cells were incubated for 30 min in PBS^++^ in absence (-) or in the presence of G-CM and processed for confocal immunofluorescence analysis using anti-LAMP2 antibody, Alexa Fluor 488-conjugated anti-mouse IgG (green), phalloidin-TRITC (red) for actin visualization and DAPI (blue) for DNA, with 63X objective. Scale bar: 15 μm. C) Schematic representation of gp90 and the recombinant protein containing the C-terminal domain of gp90 fused to GST (r-gp90). D) HeLa cell-coated microtiter plates were used for binding assays of native or r-gp90 and binding was detected in ELISA using anti-gp90 monoclonal antibody. A representative result of assays performed in triplicates, whose variation was <5%, is shown. E) HeLa cells were incubated for 30 min in PBS^++^, in absence or in the presence of 20 μg/ml r-gp90 or GST, and then processed for confocal immunofluorescence analysis as in (B). Scale bar: 15 μm. F) HeLa cells were incubated for 1 h with G strain MT in PBS^++^, in absence or in the presence of r-gp90 or GST at various concentrations, and processed as in A) for parasite counting. Values are the means ± SD of three independent assays performed in duplicate. Parasite invasion was significantly diminished in the presence of r-gp90 (*P<0.05, **P<0.0005). G) HeLa cells were incubated for 1 h with CL strain MT in D10, in absence or in the presence of r-gp90 or GST at 40 μg/ml, and processed as in (A). Values are the means ± SD of three independent assays performed in duplicate. Parasite invasion was significantly diminished in the presence of r-gp90 (*P<0.0005).

As gp90, which is released in high amounts by G strain within 1 h incubation in D10 (Fig 1A), down regulates MT invasion [7], we examined to what extent gp90 contributed to the inhibitory effect of G-CM. HeLa cells were incubated for 1 h in PBS^++^ with G strain MT in absence or in the presence of G-CM, untreated or pretreated for 15 min with anti-gp90 mAb 5E7, which does not recognize live parasites [16], or with unrelated mAb 2C2 directed to an amastigote epitope [23]. The inhibitory effect of G-CM was reversed by mAb 5E7 but not by mAb 2C2 (Fig 5A). We checked whether G-CM inhibited PBS^++^-induced lysosome scattering, by incubating HeLa cells in PBS^++^ for 30 min in absence or in the presence of G-CM. Lysosome spreading was inhibited by G-CM (Fig 5B). To ascertain that gp90 also exerted such inhibitory effect, we performed a set of experiments with the recombinant protein (r-gp90) containing the C-terminal domain of gp90 fused to GST (Fig 5C). R-gp90, which is recognized by mAb 5E7 [20], bound to HeLa cells in the same manner as the native gp90 (Fig 5D). Incubation of HeLa cells for 30 min in PBS^++^ with r-gp90, but not with GST, resulted in lysosome retention at the perinuclear area (Fig 5E), similar to that observed with G-CM (Fig 5B). G strain MT invasion of HeLa cells in PBS^++^ was inhibited by r-gp90 in a dose-dependent manner (Fig 5F). Invasion by CL was also significantly inhibited by r-gp90 at 40 μg/ml (Fig 5G).

**Fig 6:**
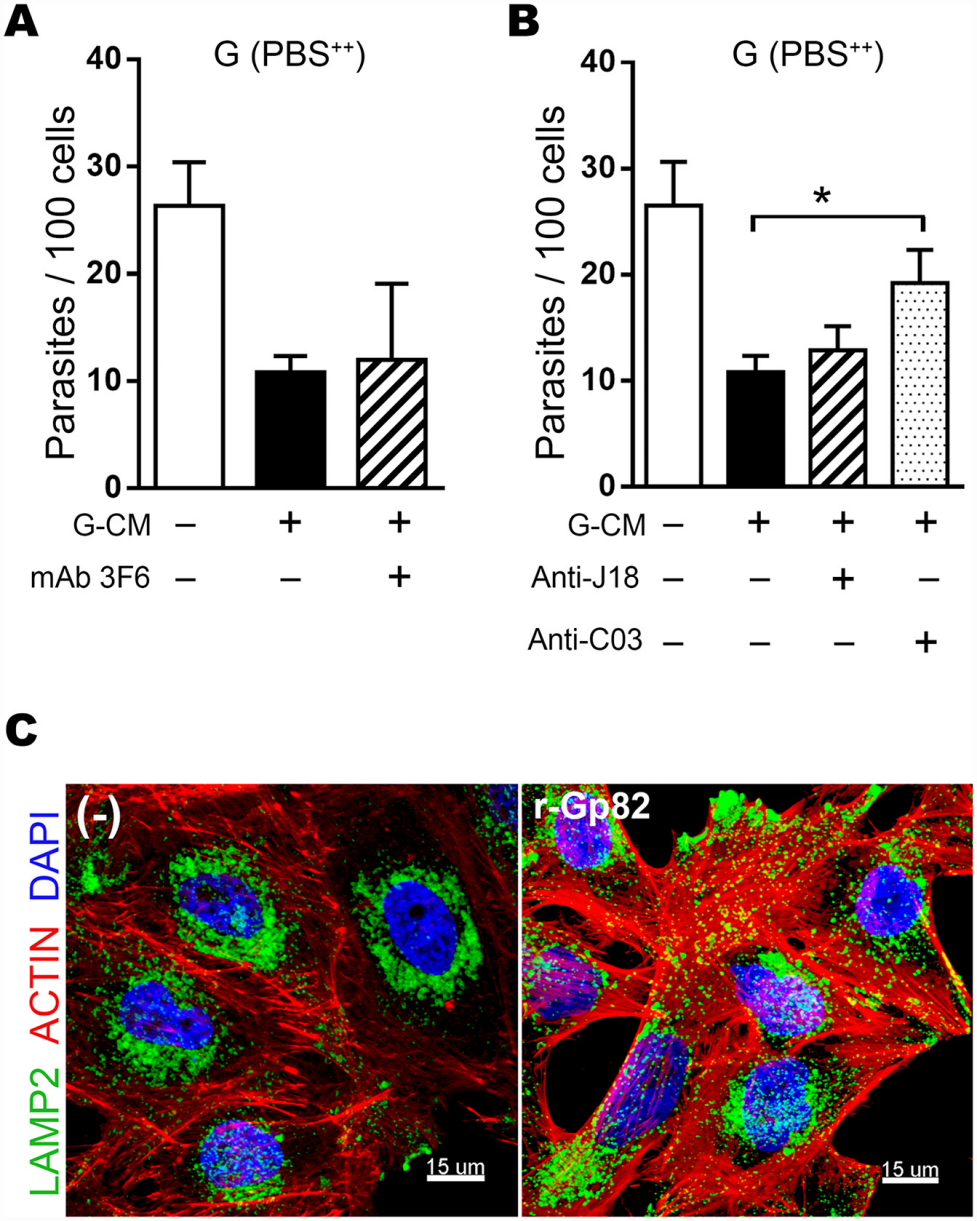
Effect of gp82 contained in G-CM on MT invasion. A-B) HeLa cells were incubated for 1 h in PBS^++^ with G strain MT in absence or in the presence of G-CM generated in D10 alone, or preincubated with anti-gp82 mAb 3F6 (A) or with polyclonal anti-gp82 antibodies (B). After fixation and Giemsa-staining, the number of intracellular parasites was counted in 250 cells. Values are the means ± SD of three independent assays performed in duplicate. The inhibitory effect of G-CM was significantly reverted by anti-gp82 polyclonal antibody anti-C03 (*P<0.005). C) HeLa cells were incubated for 30 min in absence or in the presence of 20 μg/ml recombinant gp82 (r-gp82) and processed for confocal immunofluorescence analysis as in Fig 5B.

Gp82, which has been shown to bind to target cells in a receptor-mediated manner [18,21] and to induce lysosome biogenesis [24], was also released in high amounts by G strain MT in D10 (Fig 1A). We presumed that the gp82 molecules released into medium compete with MT for the target cell receptor, thus decreasing parasite internalization, based on the findings that either the native or the recombinant gp82 (r-gp82) inhibits MT invasion [18,21]. To test to what extent the gp82 molecules present in G-CM contributed for the inhibition of MT invasion, HeLa cells were incubated for 1 h in PBS^++^ with G strain MT in absence or in the presence G-CM, untreated or pretreated for 15 min with anti-gp82 mAb 3F6. The inhibitory effect of G-CM was not reversed by mAb 3F6 (Fig 6A), possibly because the potential reverting effect of this antibody is counteracted by its inhibitory effect on MT invasion. As anti-gp82 monoclonal antibody that does not recognize live MT was not available, we tested the polyclonal anti-J18 and anti-C03 antibodies, generated in mice using respectively the recombinant protein J18, containing the full length gp82 sequence or the recombinant protein C03 with 59.1% sequence identity [18]. Both antibodies recognized the native gp82 and exhibited cross reactivity toward the recombinant proteins J18 and C03, but they differed in one important aspect: in contrast to anti-J18 antibodies, anti-C03 antibodies did not recognize live G strain MT [18]. We found that preincubation of G-CM with anti-C03 antibodies, but not with anti-J18 antibodies, partially reverted the inhibitory effect of G-CM on MT invasion (Fig 6B). The effect of the recombinant gp82 (r-gp82) in inducing lysosome spreading was also confirmed (Fig 6C) by incubating HeLa cells in the presence of 20 μg/ml r-gp82 for 30 min and then processing for confocal microscopy.

**Fig 7:**
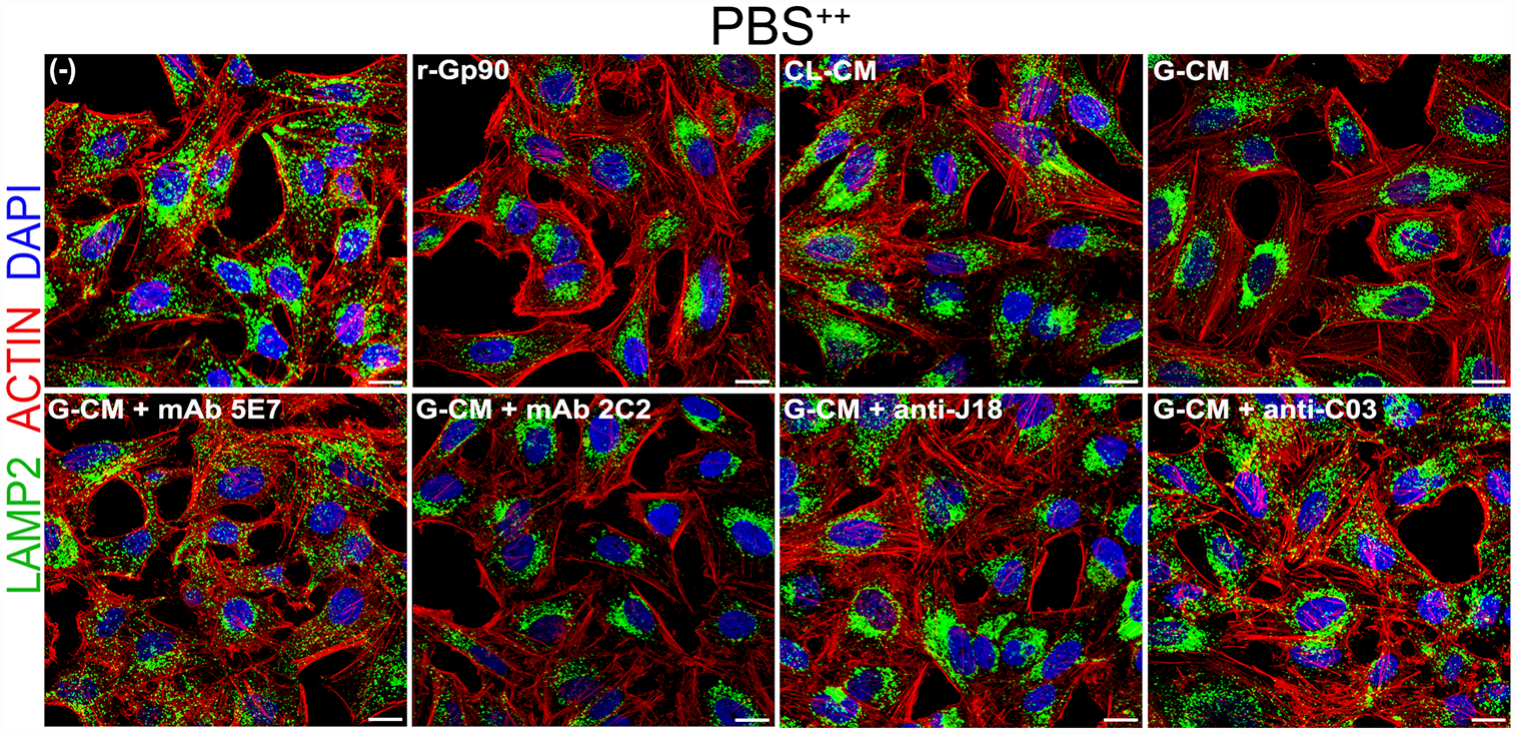
Reversal of the inhibitory effect of G-CM on PBS^++^-induced lysosome spreading by specific antibodies. HeLa cells were incubated for 30 min in PBS^++^ in absence (-) or in the presence of CL-CM, G-CM, G-CM preincubated with mAb 5E7, mAb 2C2, anti-J18 or anti-C03 antibody and processed for confocal immunofluorescence analysis using anti-LAMP2 antibody, Alexa Fluor 488-conjugated anti-mouse IgG (green), phalloidin-TRITC (red) for actin visualization and DAPI (blue) for DNA, with 63X objective. Scale bar: 15 μm. HeLa cells incubated in PBS^++^ in the presence of r-gp90 served as control for inhibition of lysosome spreading.

To determine whether the reversal of the inhibitory effect on G-CM on MT invasion by mAb 5E7 or by anti-C03 antibody (Figs 5A and 6B) was associated with the reversal of the inhibitory effect on PBS^++^-induced lysosome spreading, HeLa cells were incubated for 30 min in PBS^++^ in absence or in the presence of G-CM, untreated or pretreated for 15 min with anti-gp90 mAb 5E7, unrelated mAb 2C2, anti-J18 or anti-C03 antibody, and the spreading of lysosomes was analyzed by confocal microscopy. Inhibition of PBS^++^-induced lysosome scattering by G-CM was reversed by mAb 5E7 and by anti-C03 antibody whereas mAb 2C2 and anti-J18 antibody were without effect, the lysosomes remaining predominantly in the perinuclear region (Fig 7). In this assay, we also checked the effect of CL-CM and observed that, unlike G-CM or r-gp90, it was devoid of inhibitory effect on the PBS^++^-induced lysosome spreading (Fig 7).

### MT entry into Vero cells is inhibited by MT conditioned medium containing high gp90 and gp82 levels

To ascertain that G-CM exerted inhibitory effect on MT invasion of cells other than HeLa cells, experiments were also performed with monkey fibroblast Vero cells, following the same protocol. Vero cells were incubated for 1 h with G strain MT in PBS^++^ or with CL strain MT in D10, in absence or in the presence of G-CM generated in D10, at 1:50 dilution. The invasion rate of both strains was significantly inhibited by G-CM (S4A Fig). When G-CM preincubated with mAb 5E7 was tested, a significant reversal of the inhibitory effect was observed whereas mAb 2C2 had no such effect (S4B Fig).

## Discussion

Our results have indicated that the ability of *T*. *cruzi* metacyclic forms to enter host cells in full nutrient D10 medium is determined not only by gp90 and gp82 molecules expressed on the parasite surface but also by molecules shed into medium. The poor infective G strain MT expressed and released high levels of gp90 and gp82 in full nutrient D10 medium whereas these molecules were shed by CL strain MT in very low amounts. However, G strain MT could switch to a high invasive phenotype in PBS^++^, a nutrient-deprived medium that induces lysosome spreading and exocytosis required for the invasion process [11]. Reinforcing the idea that parasite-shed surface molecules down regulate target cell invasion, much lower amounts of gp90 and g82 were released by G strain MT in PBS^++^ than in D10. G strain MT released high amounts of gp90 and gp82 within 15 min incubation in D10, a time frame too short for the completion of MT internalization. If the release of gp90 and gp82 occurred at later time points, presumably the parasites could enter the host cells at higher rates than observed in our experiments.

Conditioned medium from *T*. *cruzi* clone Dm28c has been shown to contain large amounts of proteins [5]. This would also apply to the conditioned medium from G strain, which also belongs to the genetic group TcI [15]. From experiments with G-CM, we have inferred that, among the proteins released by G strain the most relevant for modulating parasite invasion are the gp90 and gp82 molecules. Previous studies have shown that the gp90-mediated interaction of MT with host cells is unable to trigger the signaling cascades leading to an increase in cytosolic Ca^2+^ concentration [10] that ultimately culminates in lysosome scattering/exocytosis that contributes for the parasitophorous vacuole biogenesis [11,24]. We have found that the conditioned medium of G strain MT generated in D10 inhibited the PBS^++^-induced lysosome spreading in a manner similar to the recombinant gp90. In addition, that conditioned medium exhibited an inhibitory effect on MT invasion similar to that of the recombinant gp90. A more direct evidence that gp90 contained in G-CM is involved in down regulating MT invasion was provided by experiments in which the effect of G-CM was reverted by anti-gp90 monoclonal antibody that does not recognize live MT. Despite the extensive release into medium, the levels of gp90 that remained on the parasite surface were high. We presume that the efficiency of G strain MT to invade target cells in PBS^++^ results from PBS^++^-induced lysosome scattering/exocytosis, unimpaired by gp90 molecules that are shed in smaller amounts in this medium than in D10. With regard to the gp82 molecules released into medium, they presumably compete with the MT surface gp82 for recognition by the host cell receptor. Therefore, through distinct mechanisms, both gp90 and gp82 shed by parasites exert an inhibitory effect on MT invasion.

Why G strain MT released high amounts of gp90 and gp82, as opposed to CL strain MT that do not share that property, is not known. It does not seem to be related to the differential capacity to shed vesicles. The total number of vesicles released by the two strains was similar. G strain MT released large vesicles in higher number than CL strain MT, whereas the release of smaller vesicles was the other way around. Gp90 and gp82 molecules were detected in large and small vesicles, as well as in the soluble fraction from G strain conditioned medium. With regard to mammalian cells, there are reports associating the release of extracellular vesicles with increased intracellular Ca^2+^ levels that induces cytoskeleton remodeling [25] or high expression of phospholipase D [26]. These factors may eventually have an effect on shedding of vesicles by *T*. *cruzi* MT but possibly are not determinant for the release of gp90 and gp82 molecules by CL strain MT. As a matter of fact, interaction with host cells induces in CL strain MT an increase in intracellular Ca^2+^ concentration that contributes for parasite internalization [10]. Further investigations are needed to clarify the mechanisms of differential release of gp90 and gp82 molecules by *T*. *cruzi* strains with differential ability to enter target cells.

In addition to gp90 and gp82, several other MT molecules are released into extracellular medium. Two of these molecules, SAP and TcSMP, have been shown to be involved in MT invasion of host cells [27–29]. SAP, a serine-, alanine-, and proline-rich protein expressed at higher levels in CL strain as compared to G strain, binds to host cells triggering Ca^2+^ signaling and lysosome exocytosis [27,28]. The surface membrane protein TcSMP also binds to host cells and induces Ca^2+^ response as well as lysosome spreading from the perinuclear region to the cell periphery [29]. As SAP and TcSMP proteins presumably do not interfere with gp82-binding to its receptor, it is been suggested that they act to upregulate MT invasion [27–29].

The differential ability of CL and G strain metacyclic forms to enter host cells in vitro, which is associated with the differential expression and shedding of gp90 and gp82 by these parasites, is presumably relevant for the infection of the mammalian host. Experiments performed in mice with MT of CL and G strains have revealed marked differences in their infective capacity, regardless of the mouse strain or the route of parasite inoculation [8,30,31], in close correlation with in vitro differential infectivity. Intraperitoneal or oral inoculation of CL strain MT consistently resulted in high parasitemia levels, in contrast to subpatent infection by G strain MT, which is detectable only indirectly either by xenodiagnosis or hemoculture [30,31]. Taken together, all these data reinforce the critical role played by gp90 and gp82 in MT infection.

## Supporting Information

S1 Fig Expression of surface gp90 and gp82 proteins in *T*. *cruzi* CL strain metacyclic forms after incubation in different media. Parasites were incubated or not in D10 or PBS^++^ for 1h at 37°C. After centrifugation, the supernatant was discarded and the parasites were incubated for 1 h with monoclonal antibody directed to gp90 or gp82. Following fixation and reaction with Alexa Fluor 488-conjugated anti-IgG, the parasites were analyzed by flow cytometry. Controls consisted of parasites incubated with the second antibody only.
(TIF)

S2 Fig Effect of *T*. *cruzi* conditioned medium from MT, TCT and epimastigotes on host cell invasion. A) HeLa cells were incubated for 1 h with G strain MT in PBS^++^, or with CL strain MT in D10, in absence or in the presence of G-CM from MT, TCT or epimastigote (EPI) generated in D10, and processed for intracellular parasite counting as in Fig 1B. Values are the means ± SD of three independent assays performed in duplicate. CM from and TCT and MT significantly inhibited invasion by G strain (*P<0.01, ** *P*<0.0001) and by CL strain (*P<0.05, ** *P*<0.001). B) Western blot of G-CM from MT and TCT was probed with monoclonal and polyclonal antibodies to gp90 and gp82.
(TIF)

S3 Fig Fractions of conditioned medium from CL strain and their effect on MT invasion. A) Fractions obtained from CL-CM generated in D10 or PBS^++^ were analyzed by Western blot using anti-gp82 mAb 3F6. Note the lack of detection of gp82 in fractions of CL-CM generated in PBS^++^. B) HeLa cells were incubated for 1 h with G strain MT in PBS^++^, or in PBS^++^ plus V2, V16 or vesicle-free (VF) fractions of CL-CM generated in D10 or PBS^++^. Data are representative of 3 independent experiments. No significant decrease in MT invasion was observed.
(TIF)

S4 Fig Effect of *T*. *cruzi* conditioned medium containing high gp90 and gp82 amounts on Vero cell invasion by metacyclic forms. A) Vero cells were incubated for 1 h with G or CL strain MT, in PBS^++^ or in D10, in absence of in the presence of G-CM or CL-CM generated after 1 h MT incubation in D10, and processed for parasite counting. Values are the means ± SD of three independent assays performed in duplicate. Invasion by both strains was significantly inhibited by G-CM (*P<0.005) but not by CL-CM. B) Vero cells were incubated for 1 h in PBS^++^ with G strain MT in absence or in the presence of G-CM generated in D10 alone, or preincubated with anti-gp90 mAb 5E7 or unrelated mAb 2C2, and processed for parasite counting. Values are the means ± SD of three independent assays performed in duplicate. The inhibitory effect of G-CM was significantly reverted by mAb 5E7 (*P<0.005).
(TIF)
