# Supplementary figures and images for: Surface Molecules Released by Trypanosoma cruzi Metacyclic Forms Downregulate Host Cell Invasion
Source: PLoS Negl Trop Dis. 2016 Aug 2;10(8):e0004883. doi: 10.1371/journal.pntd.0004883 (PMC4970754; doi:10.1371/journal.pntd.0004883)

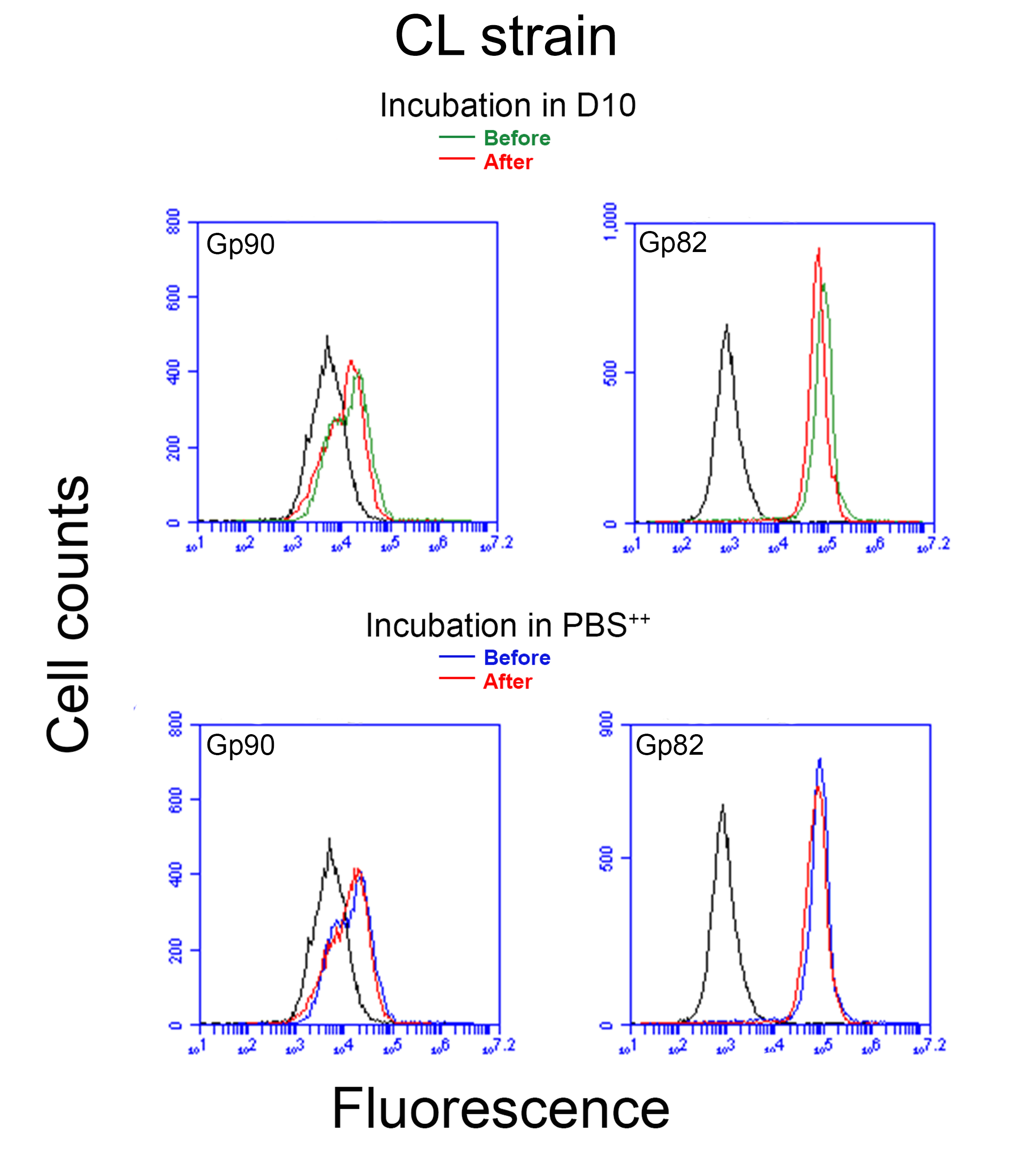

Supplement: S1 Fig — Parasites were incubated or not in D10 or PBS++ for 1h at 37°C. After centrifugation, the supernatant was discarded and the parasites were incubated for 1 h with monoclonal antibody directed to gp90 or gp82. Following fixation and reaction with Alexa Fluor 488-conjugated anti-IgG, the parasites were analyzed by flow cytometry. Controls consisted of parasites incubated with the second antibody only. (TIF) [file pntd.0004883.s001.tif]

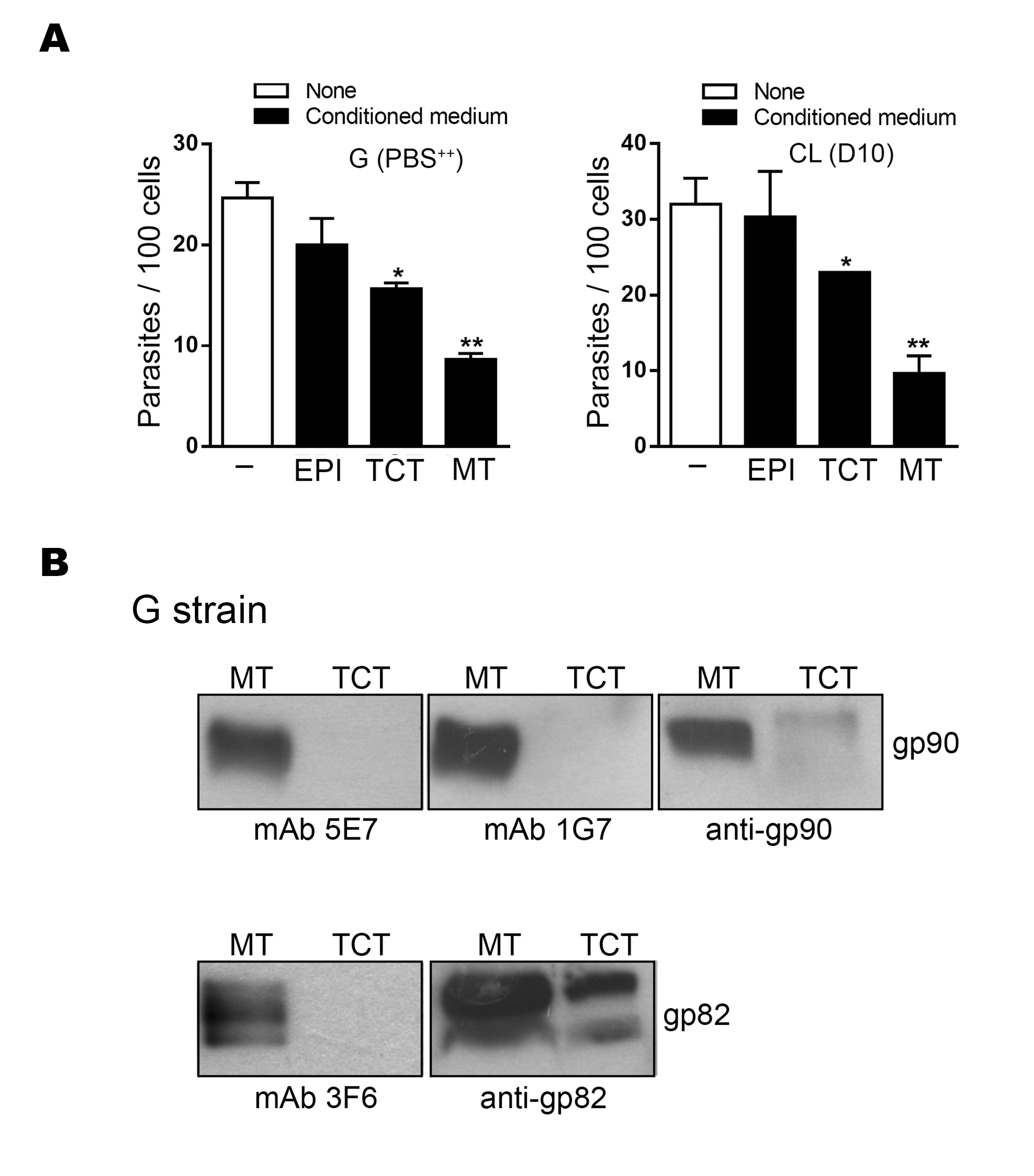

Supplement: S2 Fig — A) HeLa cells were incubated for 1 h with G strain MT in PBS++, or with CL strain MT in D10, in absence or in the presence of G-CM from MT, TCT or epimastigote (EPI) generated in D10, and processed for intracellular parasite counting as in Fig 1B. Values are the means ± SD of three independent assays performed in duplicate. CM from and TCT and MT significantly inhibited invasion by G strain (*P<0.01, ** P<0.0001) and by CL strain (*P<0.05, ** P<0.001). B) Western blot of G-CM from MT and TCT was probed with monoclonal and polyclonal antibodies to gp90 and gp82. (TIF) [file pntd.0004883.s002.tif]

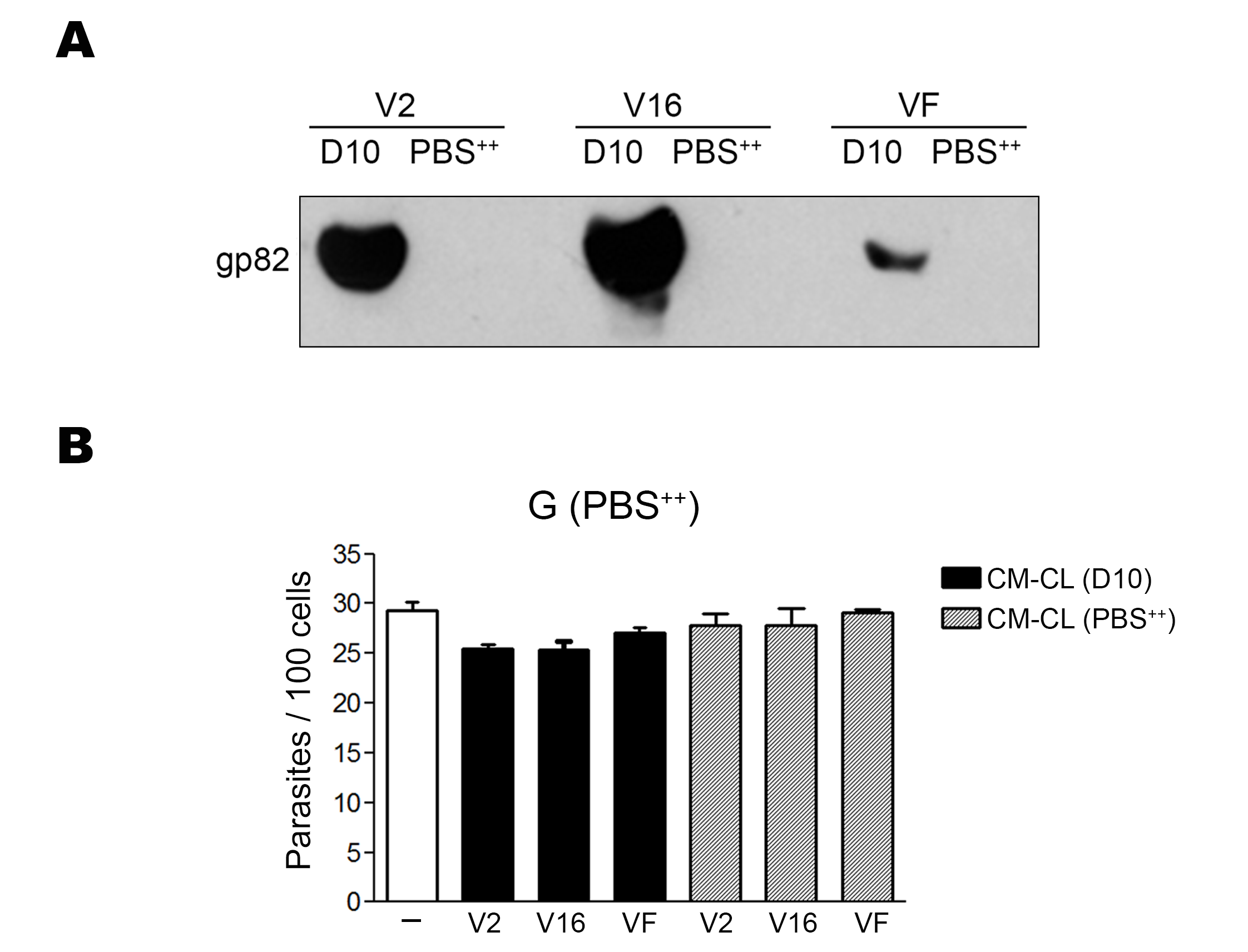

Supplement: S3 Fig — A) Fractions obtained from CL-CM generated in D10 or PBS++ were analyzed by Western blot using anti-gp82 mAb 3F6. Note the lack of detection of gp82 in fractions of CL-CM generated in PBS++. B) HeLa cells were incubated for 1 h with G strain MT in PBS++, or in PBS++ plus V2, V16 or vesicle-free (VF) fractions of CL-CM generated in D10 or PBS++. Data are representative of 3 independent experiments. No significant decrease in MT invasion was observed. (TIF) [file pntd.0004883.s003.tif]

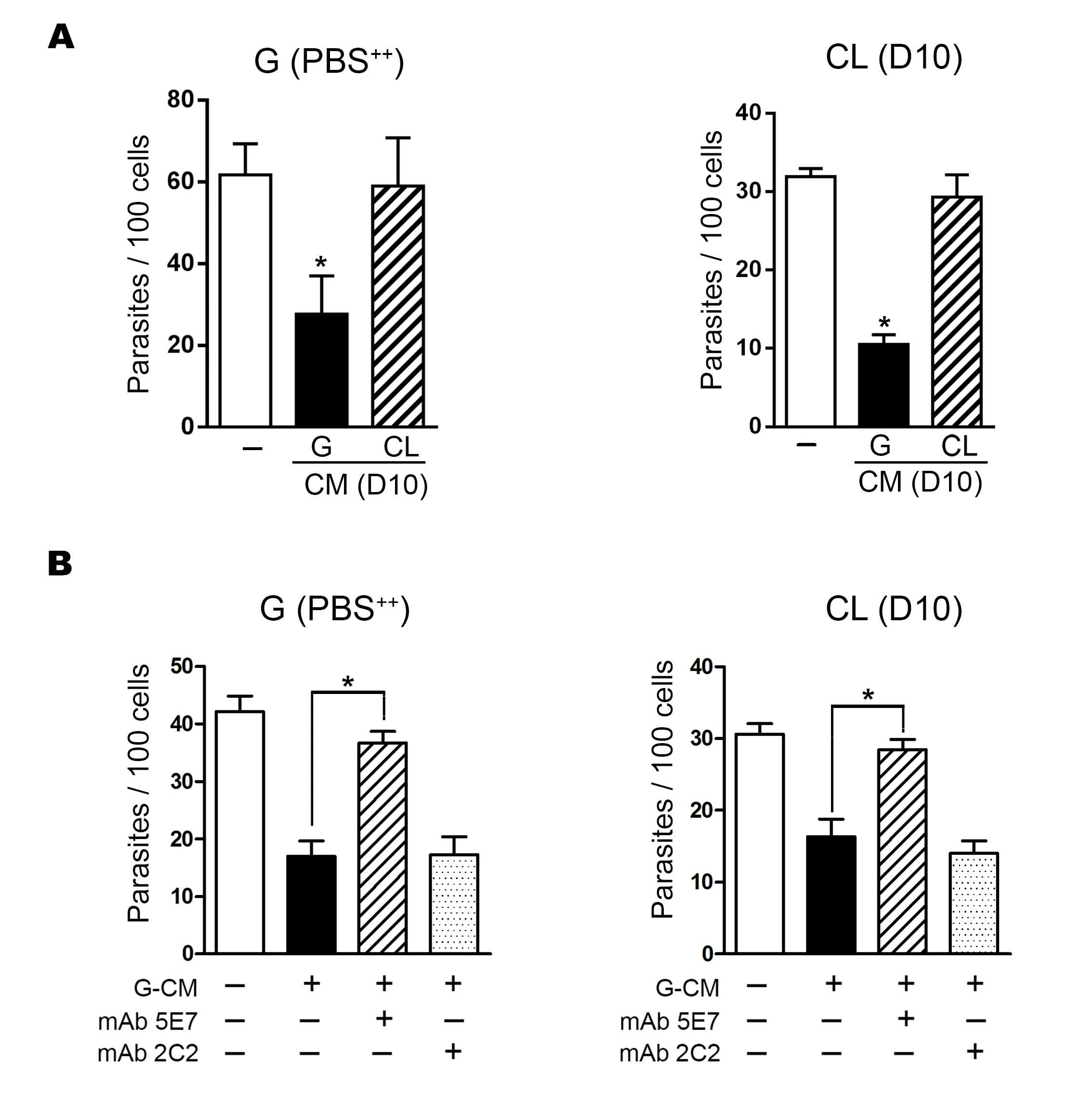

Supplement: S4 Fig — A) Vero cells were incubated for 1 h with G or CL strain MT, in PBS++ or in D10, in absence of in the presence of G-CM or CL-CM generated after 1 h MT incubation in D10, and processed for parasite counting. Values are the means ± SD of three independent assays performed in duplicate. Invasion by both strains was significantly inhibited by G-CM (*P<0.005) but not by CL-CM. B) Vero cells were incubated for 1 h in PBS++ with G strain MT in absence or in the presence of G-CM generated in D10 alone, or preincubated with anti-gp90 mAb 5E7 or unrelated mAb 2C2, and processed for parasite counting. Values are the means ± SD of three independent assays performed in duplicate. The inhibitory effect of G-CM was significantly reverted by mAb 5E7 (*P<0.005). (TIF) [file pntd.0004883.s004.tif]
